# Migrasomes, a new mode of intercellular communication

**DOI:** 10.1186/s12964-023-01121-4

**Published:** 2023-05-08

**Authors:** Yuyun Jiang, Xi Liu, Jixian Ye, Yongbin Ma, Jiahui Mao, Dingqi Feng, Xuefeng Wang

**Affiliations:** 1 Department of Central Laboratory, The Affiliated Hospital of Jiangsu University, Zhenjiang, 212001 China; 2 Department of Central Laboratory, Jintan Hospital, Jiangsu University, 500 Avenue Jintan, Jintan, 213200 People’s Republic of China; 3 Department of Nuclear Medicine and Institute of Digestive Diseases, The Affiliated Hospital of Jiangsu University, Zhenjiang, 212001 China

**Keywords:** Migrasomes, Extracellular vesicles, New mode, Intercellular communication

## Abstract

Migrasomes are newly discovered extracellular vesicles (EVs) that are formed in migrating cells and mediate intercellular communication. However, their size, biological generation, cargo packaging, transport, and effects on recipient cells by migrasomes are different from those of other EVs. In addition to mediating organ morphogenesis during zebrafish gastrulation, discarding damaged mitochondria, and lateral transport of mRNA and proteins, growing evidence has demonstrated that migrasomes mediate a variety of pathological processes. In this review, we summarize the discovery, mechanisms of formation, isolation, identification, and mediation of cellular communication in migrasomes. We discuss migrasome-mediated disease processes, such as osteoclast differentiation, proliferative vitreoretinopathy, tumor cell metastasis by PD-L1 transport, immune cell chemotaxis to the site of infection by chemokines, angiogenesis promotion via angiogenic factors by immune cells, and leukemic cells chemotaxis to the site of mesenchymal stromal cells. Moreover, as new EVs, we propose the potential of migrasomes for disease diagnosis and treatment.

Video Abstract

**Supplementary Information:**

The online version contains supplementary material available at 10.1186/s12964-023-01121-4.

## Introduction

Extracellular vesicles (EVs) are involved in various pathophysiological processes, as tools for intercellular communication have progressed rapidly. Generally, EVs are divided into three categories: exosomes, microvesicles, and apoptotic bodies [1]. Recently, migrasomes generated during cell migration have attracted considerable attention as new tools for cell communication [2]. Migrasomes are different from other EVs not only in size but also in the content and release mechanism of their vesicles [1]. Migrasomes are microscale EVs formed at the rear ends of migrating cells; similar to other EVs, they can recruit cellular contents and enrich many biomolecules, such as growth factors, cytokines, chemokines and morphogens [3]. Thus, migrasomes are thought to be new cellular messengers that release their cargo and affect the physiological and pathological processes of the surrounding cells [2]. Moreover, migrasomes appear to act as "combination signals" by simultaneously stimulating multiple key signals in target cells, thereby playing an important role in zebrafish embryonic development [3]. In this review, we summarize the formation, isolation, identification, mediation of cellular communication, and roles of migrasomes in the pathological process of diseases.

### The discovery of migrasomes

In 2015, Yu et al. discovered a new vesicle structure. The large vesicle named migrasome depends on cell migration [4, 5], the diameter of which is mostly approximately 500–3000 nm [6]. They are also called pomegranate-like structures (PLSs) because large vesicles contain various smaller vesicles and cellular contents that are shaped like pomegranates [4]. Migrasomes grow at the tips and intersections of tubular structures which are called retraction fibers (RFs) after cell migration [4], and researchers have noticed this filament in the last century [7, 8]. Ma et al. [4] discovered through time-lapse imaging and measurement and analysis of the diameter change of a single migrasome that the migrasome quickly grew to its maximum size in the initial stage and then entered a relatively stable stage. With the continuous migration of cells, the retraction fibers break, and the vesicles on them are released out of the cells or swallowed and absorbed by the next cell that reaches this position [4]. In addition, cellular contents can be actively transported into the migrasome, resulting in the increasing size of the migrasomes, which are finally released into the extracellular environment. This phenomenon still exists even though the cells gradually move away from the migrasome due to cell migration, defined as migracytosis [4]. Therefore, migrasomes mediate intercellular communication and substance transfer.

### Mechanism of migrasome formation

**Fig. 1:**
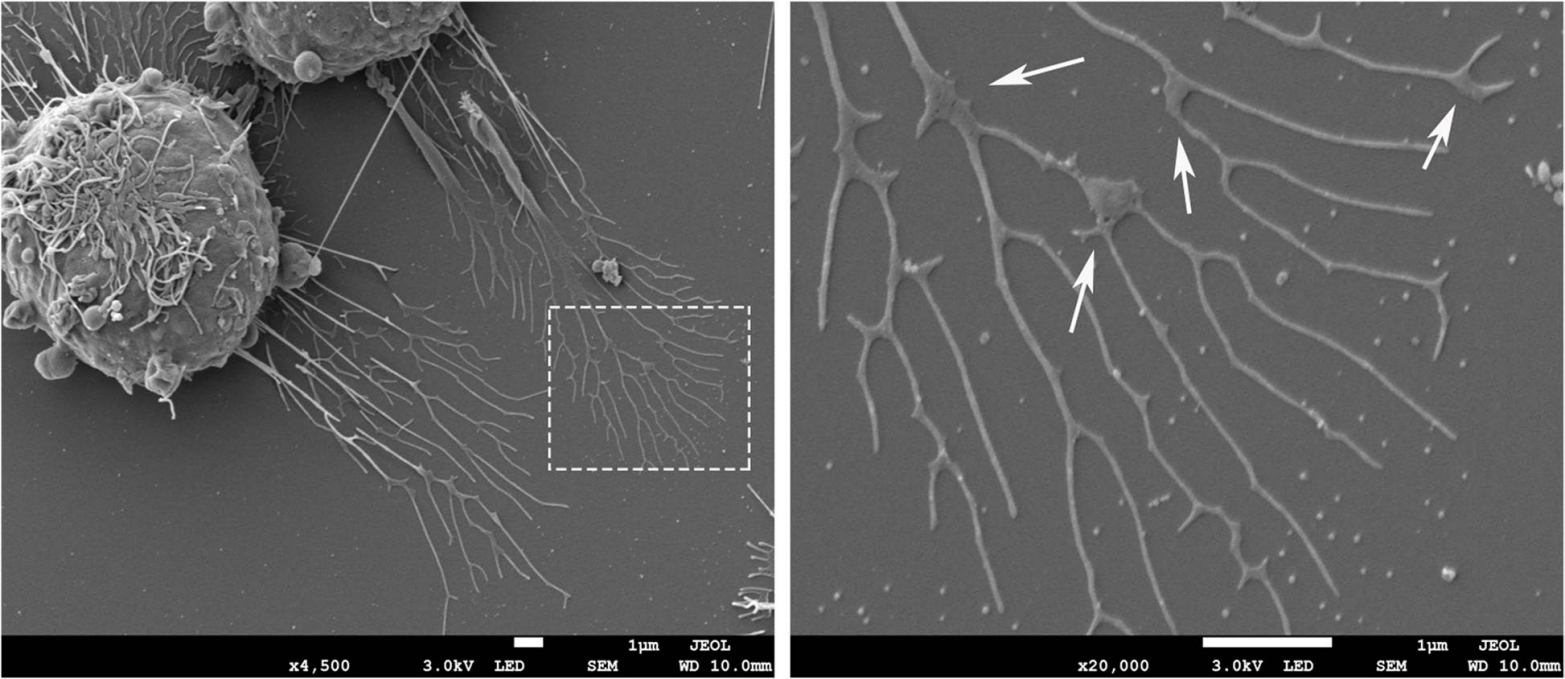
Migrasomes from RAW264.7 cells. Transmission electron microscopy image of migrasomes after 24 h of cell culture (the left side), Scale bar = 1 μm. The larger magnification image of the white box in the left (the right side), Scale bar = 1 μm. The white arrows indicate the migrasomes

Tetraspanins (TSPANs) are a family of cell-surface glycoproteins containing 33 members, the overexpression of which enhances the formation of migrasomes [9]. Tetraspanins contain four transmembrane domains that exist in almost all cell types [10]. Tetraspanin 4 (TSPAN4) is one of the most effective tetraspanins for inducing cell migration. TSPAN4 is used as a marker of migrasomes for its enrichment in migrasomes and clearest when tagged with green fluorescent protein (GFP) [9]. Various protein and lipid molecules, such as cholesterol and integrin, are recruited by tetraspanins, forming the functional and structural units of tetraspanin-enriched microdomains on the membrane. Dozens of tetraspanin-enriched microdomains constitute large domains called tetraspanin and cholesterol-enriched macrodomains (TEMAs). Huang et al. [9] measured the average fluorescence intensity of TSPAN4-GFP on migrasomes and RFs with fluorescence recovery after photobleaching and discovered that the fluorescence intensity of TSPAN4-GFP increased continuously during the growth period and became stable when the migrasome reached its maximum. Notably, the flow from the RFs to the migrasomes was irreversible. These results support the conclusion that TSPAN4 tends to accumulate in migrasomes during the growth stage. Furthermore, they used the giant unilamellar vesicle (GUV) as an in vitro system integrated with TSPAN4 and then pulled out the retraction fiber-like structures from the GUV by liquid flow pushing and manual stretching. During this process, tetraspanin-enriched microdomain gathered into the TEMAs and spontaneously formed migrasome-like structures. Moreover, migrasomes cannot be formed in the absence of TSPAN4 or cholesterol, thus verifying that TEMAs assembled by tetraspanins and cholesterol are sufficient and necessary for migrasome formation [9]. They further established a physical model to explore the mechanism by which the aggregation of TEMAs leads to the expansion of RFs into migrasomes. The concentrations of TSPAN4 and cholesterol in migrasomes far exceeded those in RFs (approximately 4–40 times), resulting in the bending rigidity of TEMAs exceeding that of other parts of the membrane, eventually leading to the expansion of RFs into migrasomes. Thus, the concentration of TSPAN4 in the membrane of the migrasome increases as the migration progresses and is essential for the stability of the mature migrasome [11]. Dharan et al. [12] recently reveal two sequential steps of migrasomes formation with live cell imaging and a biomimetic model system. The first step is driven by membrane tension, allowing the local swellings to form on the tubular retraction fibers, and then the second step is controlled by specific proteins of the TSPAN family, which make TSPAN-based membrane domains stabilize these swellings that finally turn into migrasomes. However, Gustafson et al. [13] found that chicken neural crest cells (NCCs) also produce migrasomes without TSPAN overexpression and that migrasomes provide cellular communication signals for migrating NCCs. Fan et al. [14] have verified that migrasome formation is directly linked to the persistence and speed of cell migration. We observed that RAW264.7 cells leave long RFs during migration, and migrasomes form on the tip or branching point of the RFs through transmission electron microscopy analysis (Fig. 1).

### The formation site of migrasomes

**Fig. 2:**
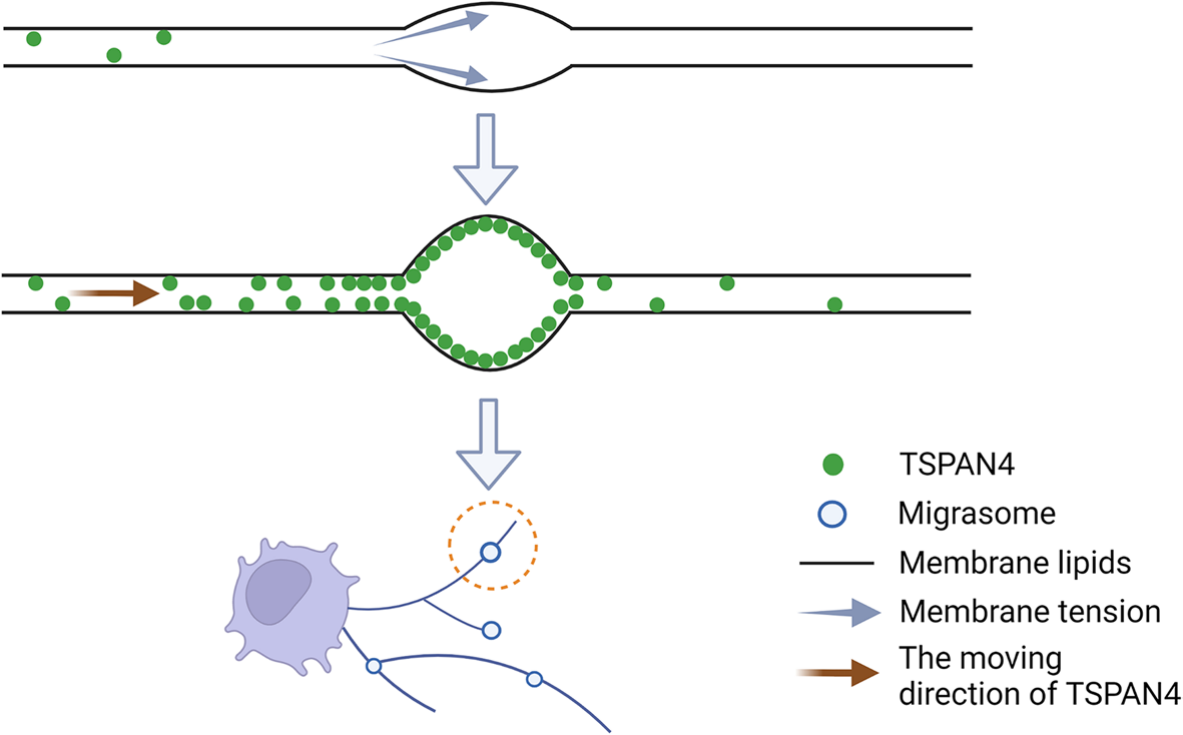
The formation of migrasomes. Membrane tension on the retraction fibers initially generates a localized swelling. Subsequently, tetraspanin-4 molecules translocate to the swelling site, triggering its expansion to several microns and culminating in the maturation of migrasomes

The process of cell migration involves not only cell morphological polarization, extending pseudopodia, and heterotopia but also requires a critical condition, that is the attachment to the surrounding environment, such as the extracellular matrix (ECM) or adjacent cells, to achieve stability [2]. The ECM is a complex molecular network structure composed mainly of intercellular substances and basement membranes [15]. The ECM is rich in collagen, fibronectin, and laminin, which can affect cell proliferation, differentiation, adhesion, and tumor metastasis [16, 17]. The integrin is primarily a cell adhesion molecule that relies on Ca^2+^ to connect cells to the extracellular environment, which mediates mutual recognition and adhesion between cells and the ECM. Integrins exist in extracellular vesicles (EVs) secreted by different cell types and can mediate the anchoring of EVs to the ECM [18, 19]. Integrins play a dual role in the formation of migrasomes. Integrins not only promote cell migration but also provide sites for migration adhesion [20]. Yu et al. first found that integrin α5β1 was enriched in migrasomes by mass spectrometry and inferred that integrin α5β1 is a fixed site of migrasome generation, which explained why migrasomes do not move along with the cell migration [20]. Fluorescent staining, time-lapse imaging and 3D distribution further showed that the integrin adhered to the bottom of the migrasome. These results further support the hypothesis that extracellular integrin fixes the site of the migrasome by adhering it to the ECM, and integrin accumulates on the spot of retraction fibers before migrasomes are formed [20]. Furthermore, they found that TSPAN4 was enriched in the head of migrasomes. These results suggest that the migrasome formation depends on tetraspanin-mediated bulbous membrane formation (especially TSPAN4) and integrin-mediated anchorage. The formation of migrasomes is shown in Fig. 2.

### The isolation and identification of migrasomes

Current research has found that a variety of cells and organs can produce migrasomes, such as blood vessels and human serum; human breast, ovarian, gastric, pancreatic, and colon cancer cells; mouse embryonic stem cells and fibroblasts; mouse eye; and human stoke specimens [2, 21]. The methods for migrasome isolation and identification have been well described in detail in Yu’s lab [22]. Similar to the isolation of EVs, migrasomes can be extracted from body fluids and conditioned cell culture media through the combination of ultracentrifugation and density gradient centrifugation [23]. Unlike the traditional method of using sucrose gradient isolation as the gold standard for EV separation, Yu’lab uses Optiprep (Sigma-Aldrich, D1556) as the density medium (https://liyu-lab-tsinghua.github.io/protocols/). In order to enhance both the yield and purity of migrasomes, similar to the innovative separation strategies of EVs [24, 25], various isolation techniques are underway. For example, Saito et al. found that cell-penetrating peptides such as pVEC and R9, and viral fusion peptide SIV promote cell migration and the formation of migrasomes [26]. Ma and collaborators demonstrated that the collected supernatant, following removal of cells and debris through centrifugation, was acquired via a 0.45-µm filter, with the resulting filtrate subsequently subjected to ultracentrifugation to pellet the migrasome-derived nanoparticles (MDNPs). The filter is inverted and squeezed by a medical syringe to collect the migrasomes [27].

As mentioned above, TSPAN4 and integrin are abundant in migrasomes, but they also appear in exosomes, making it difficult to distinguish migrasomes from exosomes by detecting TSPAN4 and integrin as markers [6]. Transmission electron microscopy (TEM) imaging is the only way to confirm migrasomes, but it is unsuitable and cannot be extended to clinical samples [6]. Chen et al. [28] found that wheat germ agglutinin (WGA) is an ideal migrasome tracker that can quickly label migrasomes, retraction fibers, and cells. In addition to the TEM imaging and WAG tracing described above [22], advances in migrasome identification have been made. For example, BODIPY ceramides can be used as an alternative or additional stain for migrasomes, such as WGA [13]. Zhu et al. [29] used the nucleic acid stain SYTO14 as a secondary marker of the migrasome to label the migrasome mRNA. Jing et al. [30] developed fluorescent artificial antigens (FAAs) based on quantum dots coated with polyacrylic acid. The expanded membrane network from living DC2.4 dendritic cells (DCs) was captured by tracking with a single FAA-labeled quantum dot particle, and the DC cell membrane fiber and migrasome morphology were characterized by ultrahigh resolution. The migrasome is rich in many protein markers, such as carboxypeptidase Q (CPQ), EGF domain-specific O-linked N-acetylglucosamine transferase (EOGT), bifunctional heparin sulfate N-deacetylase/N-sulfotransferase 1 (NDST1), and phosphatidylinositol glycan anchor biosynthesis, class K (PIGK). These protein markers can be used to distinguish between migrasomes and EVs [22, 31]. Liu et al. [32] found that migrating podocytes expressed high levels of miR-1303, miR-490-5p, miR-548a, miR-611, and miR-661, while podocyte exosomes expressed high levels of miR-144-3p, miR-221-3p, and miR-4286. Podocyte damage increases the release of migrasomes, and the number of migrasomes in the urine is a sensitive indicator of early podocyte injury. However, the role of these specific miRNAs carried by migrasomes from podocytes in cell communication was not examined.

### Migrasomes mediate cell-to-cell communication

Migrasomes are rich in signaling molecules such as cytokines, chemokines, and growth factors, which can be captured by surrounding cells and affect and change the behavior and state of recipient cells. Migrasomes can also remain on the migration path of migrating cells, acting as location signals after the cells have been away. Thus, migrasomes can integrate biological and spatial signals, deliver combined signals, and mediate cell-to-cell communication. For example, Jiang et al. [3] found a large number of migrasomes and retraction fibers in zebrafish embryos. After knocking out TSPAN4a and TSPAN7, key genes for migrasome formation, migrasomes are reduced, and asymmetric developmental defects occur during the zebrafish gastrulation [3]. Mechanistically, mesodermal and endodermal cells produce CXCL12-rich migrasomes. CXCL12 in migrasomes interacts with CXCR4, the ligand expressed on dorsal forerunner cells (DFCs), to induce chemotaxis and recruit DFCs, thereby ensuring the correct positioning of DFCs and subsequent organ morphogenesis [3]. Hyun et al. [33] also found membrane-covered structures and vesicles formed by fibers in the migration path of neutrophils. This membrane-covered structure also releases CXCL12 and guides T cells to follow the path of neutrophil migration. Granular-specific CXCL12 depletion and CXCR4 antagonism cancel neutrophil guidance and T-cell migration [34, 35]. Thus, chemokine-rich migrasomes not only orchestrate embryonic origin but also direct immune cell migration to exert effects.

Jiao et al. [36] found that damaged mitochondria could be transported into migrasomes and subsequently discarded from migrating cells under mild mitochondrial stress. Mechanistically, mitocytosis locates damaged mitochondria at the cell periphery, avoiding the binding of damaged mitochondria to inward motor proteins (dynein) and enhancing their binding to outward motor kinesin 1 (KIF5B). The damaged mitochondria are then transported to the migrasomes where they are disposed. Macrophages and neutrophils produce migrasomes. However, knockout of the TSPAN9 gene reduces the production of migrasomes, mitochondrial membrane potential (MMP), and cell viability and migration. Thus, migrasomes can be used as a disposal mechanism for damaged organelles to maintain cell homeostasis and quality control.

**Fig. 3:**
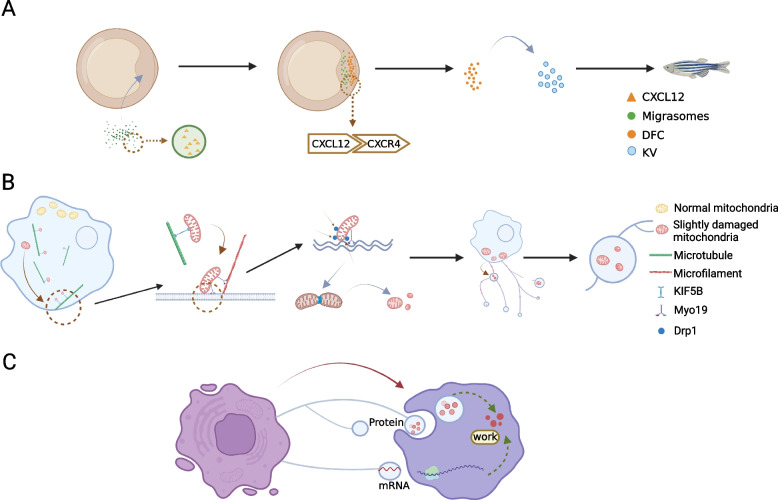
Migrasomes mediate cell-to-cell communication. **A** The effect of signaling molecules carried by migrasomes on the host: migrasomes release CXCL12, attract DFCs to gather on the embryo shield, and promote the asymmetric development of zebrafish organs. **B** Migrasomes release slightly damaged mitosomes to maintain intracellular mitochondrial homeostasis. **C** Protein and mRNA are transferred to receptor cells by migrasomes to work

Similar to other EVs, migrasomes can transfer cell contents to surrounding cells. Zhu et al. [29] demonstrated that PTEN is one of the richest mRNAs in migrasomes, purified migrasomes are added to recipient tumor cells, and PTEN protein can be translated into recipient cells, thereby reducing pAKT activity and the proliferation of tumor cells. These results suggest that migrasomes can transfer cellular content and alter the activity and function of recipient cells. Migrasomes are new tools for cell-to-cell communication. Migrasome-mediated cell communication is shown in Fig. 3.

## Migrasomes mediate the pathological process of diseases

At present, most research focuses on the basic biology of migrasomes, especially their discovery, generation, characteristics, and functions. The role of migrasomes in pathophysiological processes remains to be determined, but growing evidence suggests that migrasomes are involved in the development and evolution of many diseases. For example, high-salt diet induces microglia/macrophages to produce migrasomes, which leads to neuronal loss after stroke [37]. Podocyte injury produces migrasomes, which can be detected in the urine of mice with puromycin aminonucleoside (PAN)-induced nephropathy and patients with diabetic nephropathy [32]. Platelets infected with severe acute respiratory syndrome coronavirus-2 (SARS-CoV-2) virions release migrasomes, which mediate immune dysregulation and thrombosis [38]. TNF-α-activated primary human coronary artery endothelial cells (pHCAECs) can release migrasomes, which mediate the signal transmission between migrating pHCAECs, coordinating the direction and parallel movement of cells during migration [39]. The role of migrasomes in the above diseases has been reported by Zhang [2], but the function of migrasomes in a variety of pathophysiology has been gradually discovered and gain attention.

Osteoclasts (OCs), originating from monocytes/macrophages, play a key role in bone development, bone remodeling, and fracture healing [40, 41]. Lampiasi et al. [42] studied the timing and performance of OC differentiation stimulated by RANKL and found migrasome-like vesicles along filamentous pseudopods when mononuclear preosteoclasts approached and fused with other preosteoclasts. Migrasomes provide spatiotemporal information for cellular communication during cell migration [4]. These migrasomes generated during mononuclear pre-OC migration may also mediate the migration and fusion of pre-OCs and subsequent OC differentiation [42].

Retinal pigmented epithelium (RPE) activated by cytokines can lead to proliferative vitreoretinopathy (PVR), causing retinal detachment and blindness [43, 44]. Wu et al. [45] found that human PVR samples express the migrasome marker, TSPAN4. RPE from Norway rat activated by TGF-β1 increases TSPAN4 expression and produces migrasomes. Moreover, RPE can internalize migrasomes and enhance their migration and proliferation. Mechanically, TGF-β1/Smad2/3 signaling is involved in TSPAN4 expression and migrasome production in rat RPE. Thus, migrasomes play a key role in PVR progression induced by RPE activation.

Given that migrasomes are formed during cell migration, they may play an important role in tumor metastasis and immune disorders because of the high migration of tumor cells and immune cells. Wang et al. [46] found that PD-L1 is abundant in the rear of migrating cancer cells, forming PD-L1-enriched retraction fibers and migrasomes. PD-L1-rich migrasomes may be internalized by neighboring cells and increase the expression of PD-L1, thereby inhibiting the immune response of individuals, and may secrete chemokines that promote the migration of tumor and stromal cells in the tumor microenvironment. Migration of tumor cells produces migrasomes, and the cytoplasmic content in migrasomes is endocytosed by the surrounding cells, which is one of the mechanisms of tumor metastasis. The use of nanoparticles to cover the surface of migrasomes, inhibit the uptake of migrasomes by surrounding cells, and suppress tumor cell metastasis is a powerful strategy for anti-metastasis in tumor treatment [47].

Exosomes in extracellular vesicles have been widely studied and are involved in cancer metastasis [48–50]. Metastatic cancer cells produce exosomes that induce epithelial-mesenchymal transformation (EMT) of surrounding cells [51], affecting distant cell types to modulate premetastatic niches [52], thereby altering the migration behavior of neighboring cells [19, 53–55]. Migrasomes can also deliver biomolecular cargo, such as exosomes. In addition to the aforementioned PD-L1-rich tumor cell migrasomes [46], tumor cell migration may also release migrasomes rich in proangiogenic factors or metalloproteinases to promote tumor cell infiltration and metastasis. Moreover, migrasomes are rich in cytokines and chemokines [22], and many immunosuppressive cells or molecules can be driven by migration to the tumor microenvironment to mediate the immune escape of tumor cells.

As the most potent antigen-presenting cells, dendritic cells (DCs) play a vital role in innate and adaptive immune responses [56, 57]. Jing et al. [30] observed migrasomes in the dendritic cell membrane fiber network of DC2.4 cells. They suggested that DCs might use these specialized vesicles to transfer antigens and chemokines or as a means of intercellular communication to mediate linkage with other DCs. These cellular components may be partially processed or present in migrasomes. Neutrophils are abundant innate immune cells that play an important role in inflammation, infection, and tissue remodeling. Lim et al. [34] found that migrating neutrophils left a membrane-covered structure to release CXCL12, which guided CD8^+^ T cells to migrate to the influenza-infected trachea. The membrane-covered structures produced during neutrophil migration are now referred to as migrasomes. Indeed, mouse neutrophils can form migrasomes containing mitochondria with damaged membrane potential in vivo, suggesting that migrasomes may be a way to remove damaged mitochondria and maintain organelle homeostasis [36]. Bao et al. [58] found that circulating neutrophils carried mitochondria-rich EVs to improve endothelial dysfunction in mice with sepsis. The formation of these EVs relies on TSPAN9 and is morphologically similar to the migrasomes produced via mitocytosis. In addition to desposing damaged mitochondria through migrasomes [36], neutrophils also transport superoxide dismutase 2 (Sod2) through migrasomes to mediate antithrombosis, maintain endothelial homeostasis, and protect the host against sepsis [58]. Similarly, immune cells can be driven to sites of infection or inflammation by cytokine- and chemokine-rich migrasomes, exerting pro-inflammatory or anti-inflammatory effects and exacerbating or mitigating the disease response.

Recently, Zhang et al. [59] found that monocytes produce migrasomes to promote capillary formation in chicken embryos. Monocyte depletion reduces the number of migrasomes and weakens the formation of capillaries. Mechanically, monocytes produce migrasomes rich in VEGFA and CXCL12, which promote capillary formation and monocyte recruitment in the chorioallantoic membrane of chick embryo. Thus, monocytes promote angiogenesis using migrasomes rich in pro-angiogenic factors. The role of monocytes and macrophages in promoting angiogenesis has been demonstrated in tumor and peripheral nerve regeneration [60, 61]. For example, monocytes can migrate inside tumors due to attraction to chemokines, where they mature into tumor-associated macrophages (TAMs). TAMs can secrete a variety of cytokines and growth factors to promote angiogenesis [62, 63]. Macrophages secrete VEGFA, which induces angiogenesis to relieve transected sciatic nerve hypoxia. Schwann cells then carry regenerative axons across the transected region using neovascularization as a "track" and promote peripheral nerve regeneration [64]. Whether monocytes and macrophages use migrasomes to promote angiogenesis in tumors and peripheral nerve regeneration needs further study. However, the use of migrasomes by these cells to promote angiogenesis provides important insights into tumor progression and nerve regeneration.

Mesenchymal stromal cells (MSCs), multipotent nonhematopoietic stem cells with self-renewal capability, are components of the bone marrow stem cell niche and promote hematopoiesis in the bone marrow microenvironment [65, 66]. Deniz et al. [67] found that MSCs produce migrasomes which contain signaling molecules such as stromal cell-derived factor 1 (SDF-1, often referred to as CXCL12) that facilitate the migration of hematopoietic cells like KG-1a cells and primary CD34^+^ hematopoietic stem.and progenitor cells (HSPCs). MSC-associated migrasomes may play an important role in exchanging information between cellular components of the bone marrow microenvironment, because migrasomes are selectively taken up by migrating leukemic cells. Whether MSCs guide cancer cells to migrate and/or reside in the bone marrow via migrasomes, thus forming potential targets for cancer therapy, remains to be further evaluated.

**Fig. 4:**
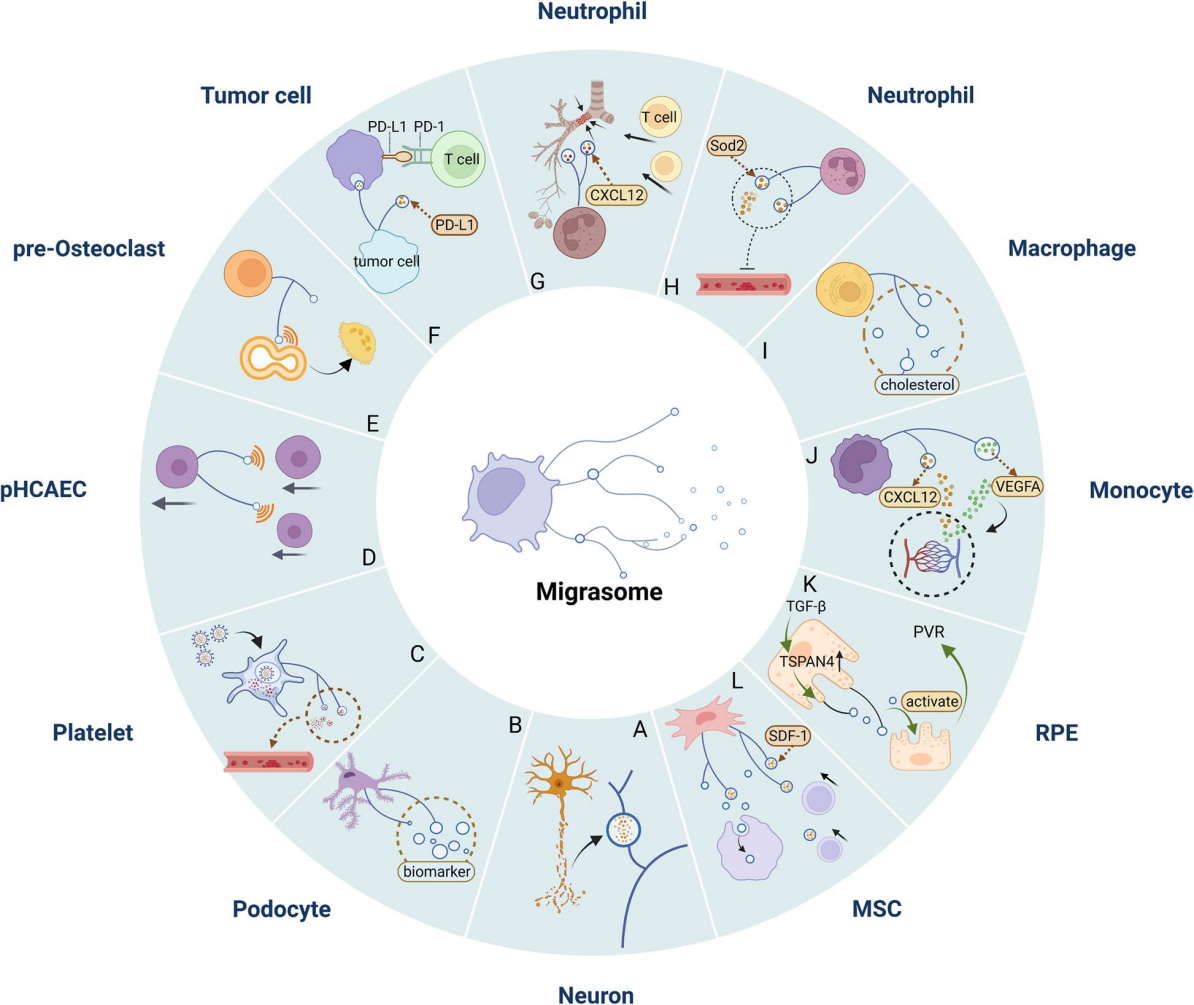
Migrasomes mediate the pathological process of diseases. **A** Migrasomes incorporate and deliver cytosol from surrounding damaged neurons. **B** Migrasomes serve as a biomarker of podocyte damage. **C** Platelets internalize SARS-CoV-2 and release migrasomes containing the contents of platelets, resulting in thrombosis. **D** Migrasomes released by pHCAECs secrete signals to guide the direction of other pHCAECs. **E** Migrasomes released by pre-OCs mediate the migration and fusion of other pre-OCs and subsequent OC differentiation. **F** Migrasomes containing PD-L1 released by tumor cells are internalized by surrounding cells, increase the expression of PD-L1 and inhibit the activity of immune cells. **G** Migrasomes containing CXCL12 released by neutrophils guide T cells to migrate to the influenza-infected trachea. **H** Migrasomes transport substantial Sod2 and serve as antithrombotic mediators. **I** Macrophages secrete cholesterol by migrasomes and retraction fibers. **J** Migrasomes generated by monocytes deliver VEGFA and CXCL12 to the area of capillary formation to provide a favorable microenvironment for angiogenesis. **K** Migrasomes modulated by overexpressing TSPAN4 or stimulating RPE with TGF-β play an important role in the activation of RPE and progression of PVR. **L** MSC-associated migrasomes containing SDF-1 attract leukemic cells to migrate and can be taken up by leukemic cells

In addition, the shedding of cholesterol-rich microbubbles might be related to cell migration when new biological imaging methods are used to deeply understand the pathways and mechanisms of cholesterol efflux. Since the rich cholesterol in migrasomes and membrane fragments can be released in the form of vesicles and nanotubes during cell migration [68], migration of migrasomes from cells may contribute to cholesterol efflux [69]. Macrophage cholesterol efflux was expected to delay the formation of macrophage foam cells in atherosclerotic plaques. Most studies have suggested that macrophage cholesterol efflux is primarily due to the action of ABC transporters [70, 71], which export cholesterol to high-density lipoprotein (HDL) [72]. However, He et al. [73] found that excess cholesterol from macrophages could be transferred directly to cocultured smooth muscle cells without dependence on ABCA1. However, whether macrophages transfer surplus cholesterol through shed microvesicles or migrasomes requires further study. The study of migrasome-mediated pathological processes in diseases has just begun, and many questions await answers. The migrasome-mediated pathological process of the diseases is shown in Fig. 4.

## Summary and prospects

The migrasome is a new EV that is associated with cell migration. The retraction fibers at the rear of the cells are stretched to form a large vesicular structure, approximately 500–3000 nm in size, called the migrasome during cell migration. In addition to containing proteins, mRNA, and miRNAs similar to other EVs, migrasomes also contain many small vesicles and damaged mitochondria [2]. Although the migrasome can rapidly recruit cellular contents, such as TSPAN4 moving into the migrasomal membrane by retraction fibers, the sorting and transporting mechanism of small vesicles, proteins, nucleic acids, and other contents into migrasomes remains unclear. The abundance of enzyme proteins, such as CPQ, EOGT, NDST1, and PIGK [22, 31], suggests a selective and unique cargo recruitment mechanism for migrasomes, but this still needs to be further confirmed.

EVs carry proteins and nucleic acids from their parental cells and can be used for early diagnosis of a variety of diseases [74–76]. For example, circulating microvesicles can be used as biomarkers for the diagnosis of cardiometabolic diseases [77]. MicroRNA analysis of urine and brain EVs can serve as an early diagnostic tool in patients with prostate cancer and traumatic brain injury, respectively [78–80]. Migrasomes can also transport cytosolic contents, and the number of migrasomes in urine is a biomarker of early podocyte injury [32]. Moreover, the expression profiles of miRNAs in migrasomes and exosomes from damaged podocytes are different; however, the relationship between miRNA expression and podocyte injury has not been confirmed [32]. Furthermore, similar to the distribution of EVs in biological fluids, migrasomes are also distributed in serum and urine. EV-based testing combined with modern diagnostic techniques can improve test specificity. For example, the ExoDx Prostate (IntelliScore) based on a urine exosome gene expression assay is superior to tissue biopsy and is a noninvasive diagnostic method for diagnosing high-grade prostate cancer [81]. Therefore, it is believed that migrasomes can also be added to the list of diagnostic EVs, although this avenue is still long due to the infancy of migrasome research.

Neutrophils, macrophages, and DCs are innate immune cells that can release migrasomes, and the migrasomes released by neutrophils can guide CD8^+^ T cells to the site of pathogen infection [34], indicating that migrasomes can guide and exert immune responses. It is well known that, in addition to secreting cytokines and chemokine interactions, immune cells can also communicate with each other through EVs to regulate immune responses [1, 82]. EVs carry cellular content and promote immune responses. For example, DC- and macrophage-derived exosomes can induce protection against *Toxoplasma gondii* and *Mycobacterium tuberculosis* in mice, respectively [83, 84]. Tumor cell-derived exosomes can activate a variety of antitumor immune responses, including the activation of CD8^+^ T cells [85], NK cells [86], macrophages [87], and helper T cells [88]. Migrasomes can also interact with immune cells through cytokines and chemokines. Moreover, unlike the unoriented release of EVs, migrasomes can guide immune cells in the correct path and direction, such as the aforementioned neutrophil-released migrasomes that direct CD8^+^ T cells to the site of influenza infection [34]. Cell migration is a basic phenomenon, particularly the migration of immune cells to sites of infection or inflammation. Therefore, how immune cells use the guidance of migrasomes to exert anti-infection or antitumor effects is a very attractive direction, although there is a long way to go to clarify the roles and functions of migrasomes in a variety of pathophysiological processes.

Similar to other EVs, cargo information in migrasomes can stimulate recipient cells and change their signaling pathways. For example, PTEN and PD-L1 in migrasomes affect the activity of pAKT and expression of PD-L1 in recipient tumor cells, respectively [29, 46]. However, cargo packaging, transportation, and effects on recipient cells by migrasomes are still in their infancy. Further basic and clinical research will contribute to understanding this fascinating vesicle of the migrasome in the future.

## Conclusion

In recent years, microscale migrasomes have opened a new vision as a unique type of extracellular vesicles. Although their characteristics and functions have been gradually elucidated, the biogenesis mechanism of migrasomes, especially in pathophysiology, is still in its infancy. In this review, we highlighted the messenger role of migrasomes in various pathophysiology and proposed their potential in disease diagnosis and treatment. However, the mechanisms of migrasome formation remain incomplete. Efforts are currently needed to be directed towards elucidating the mechanisms underlying the packaging of cytosolic proteins into migrasomes, as well as characterizing the signaling molecules that are differentially enriched in these migrasomes derived from distinct cell types. Understanding the specific roles played by such molecules in various physiological and pathological contexts is a major objective of this line of investigation. In addition, current available separation methods of migrasomes are difficult to ensure the purity and yield of migrasomes. Repeatable isolating homogeneous migrasomes from different batches is the crucial factor to explore their functions. Overall, more insights into the pathophysiological role of migrasomes may advance modern EV-based diagnosis and treatment, especially when the mechanism of intercellular communication by migrasomes are further explored and revealed in various pathophysiological conditions.

## Data Availability

Not applicable, please refer to the original references.

## Abbreviations

EVs: Extracellular vesicles
TEM: Transmission electron microscopy
TSPAN4: Tetraspanin 4
ITGA5: Integrin alpha 5
ECM: Extracellular matrix
TEMAs: Tetraspanin and cholesterol-enriched macrodomains
RFs: Retraction fibers
GUV: Giant unilamellar vesicle
NCCs: Neural crest cells
CPQ: Carboxypeptidase Q
EOGT: EGF domain-specific O-linked N-acetylglucosamine transferase
NDST: Bifunctional heparin sulfate N-deacetylase/N-sulfotransferase 1
PIGK: Phosphatidylinositol glycan anchor biosynthesis, class K
CXCL12: Chemokine ligand 12
CXCR4: Chemokine receptor 4
DFC: Dorsal forerunner cell
KV: Kupffer's vesicle
KIF5B: Recombinant kinesin family, member 5B
Myo19: Myosin XIX
Drp1: Dynamin-related protein 1
SARS-CoV-2: Coronavirus-2
pHCAEC: Primary human coronary artery endothelial cell
OC: Osteoclast
PD-L1: Programmed cell death-ligand 1
PD-1: Programmed death 1
Sod2: Superoxide dismutase 2
VEGFA: Vascular endothelial growth factor A
TGF-β: Transforming growth factor-β
RPE: Retinal pigmented epithelium
PVR: Proliferative vitreoretinopathy
TAMs: Tumor-associated macrophages
DCs: Dendritic cells
WGA: Wheat germ agglutinin
MSC: Mesenchymal stromal cells
SDF-1: Stromal cell-derived factor 1
